# More indications for redox-sensitive cysteine residues of the Arabidopsis 5-aminolevulinate dehydratase

**DOI:** 10.3389/fpls.2023.1294802

**Published:** 2024-01-22

**Authors:** Daniel Wittmann, Chao Wang, Bernhard Grimm

**Affiliations:** ^1^ Institute of Biology/Plant Physiology, Humboldt-Universität zu Berlin, Berlin, Germany; ^2^ State Key Laboratory of Crop Stress Adaptation and Improvement, School of Life Sciences, Henan University, Kaifeng, China

**Keywords:** 5-aminolevulinic acid, HEMB, tetrapyrrole biosynthesis pathway, redox control, chlorophyll biosynthesis, thioredoxin, thiol switch, chloroplast biogenesis

## Abstract

Redox-dependent thiol-disulfide switches of cysteine residues are one of the significant posttranslational modifications of proteins to control rapidly their stability, activity, and protein interaction. Redox control also modulates the tetrapyrrole biosynthesis (TBS). Among the redox-dependent TBS enzymes, 5-aminolevulinic acid dehydratase (ALAD) was previously recognized to interact with reductants, such a thioredoxins or NADPH-dependent thioredoxin reductase C. In this report, we aim to verify the redox sensitivity of ALAD and identify the redox-reactive cysteine residues among the six cysteines of the mature protein form Arabidopsis. Based on structural modelling and comparative studies of wild-type ALAD and ALAD mutants with single and double Cys➔Ser substitutions under oxidizing and reducing conditions, we aim to predict the dimerization and oligomerisation of ALAD as well as the crucial Cys residues for disulfide bridge formation and enzyme activity. The Cys404Ser mutation led to a drastic inactivation of ALAD and redox-dependent properties of ALAD were severely impaired, when Cys71 was simultaneously mutated with Cys152 or Cys251. Cys71 is located in a flexible N-terminal arm of ALAD, which could allow intramolecular disulfide bridges with Cys residues at the surface of the remaining globule ALAD structure. As a result, we propose different roles of Cys residues for redox control, catalytic activity and Mg^2+^-dependent assembly.

## Introduction

Chlorophyll is the world’s most abundant pigment and essential for oxygenic photosynthesis. It is assembled in the pigment-binding proteins of the core and light-harvesting complexes of photosystems I and II (PSI and PSII) and absorbs light for the downstream energy conversion through the photosynthetic electron transport chain. The final steps of chlorophyll synthesis in angiosperms require a tight and light-dependent control due to the exclusively light-operating protochlorophyllide oxidoreductase (POR). Moreover, the synchronized synthesis of chlorophyll and nuclear and plastid-encoded chlorophyll-binding proteins ensures the supply of stoichiometric amounts of chlorophyll for the direct association with the pigment-binding proteins. As an additional level of regulation, posttranslational control mechanisms of enzymes in chlorophyll biosynthesis allow a rapid response to the continuously changing environmental conditions, for example, the daily varying light intensities and temperatures.

The redox-dependent control of proteins is based on the thiol-disulfide changes of cysteine residues (Cys) and belongs to the stringent posttranslational regulatory mechanisms, which maintain stability, catalytic activity or protein-protein interactions in multiple cellular processes, including the metabolic pathway of chlorophyll biosynthesis (Wittmann et al., 2020). Photosynthetic electron transport provides a reducing environment in chloroplasts, making electrons available for many metabolic pathways. Reduced ferredoxin provides electrons for the thioredoxin (TRX)-driven redox machinery via ferredoxin-thioredoxin reductase (FTR; Buchanan and Balmer, 2005; Dai et al., 2007)., Among others in plastids, electrons can also be transferred through the NADPH-dependent thioredoxin reductase C (NTRC) to 2-cysteine peroxiredoxin (2-CP), to detoxify also hydrogen peroxide (H_2_O_2_, Serrato et al., 2004; Toivola et al., 2013; Pérez-Ruiz et al., 2017; Cejudo et al., 2019). It was previously thought that NTRC also contribute to the reduction of enzymes of chloroplastic primary metabolism (Moon et al., 2006; Thormählen et al., 2013; Pérez-Ruiz et al., 2014). The more recent proposal by Perez-Ruiz et al. is based on the analysis of ntrc mutants, according to which the altered redox states of plastidic enzymes are not primarily explained by the absence of NTRC, but by the lack of reducing power of TRX isoforms, which complementarily substitute the absence of NTRC for the reduction of 2-CP, thus neglecting the enzymes that are the actual TRX-dependent targets (Perez-Ruiz et al., 2017).

Among the TBS enzymes, 5-aminolevulinic acid dehydratase (ALAD), also known as porphobilinogen synthase (PBGS), was identified as an interaction partner of NTRC, f- and m-type TRXs by pulldown and bimolecular fluorescence complementation (BiFC) assays (Wittmann et al., 2018). These findings confirmed previous results, which were initially obtained through different proteomic screens of protein extracts from *Synechocystis* PCC6803 (Lindahl and Florencio, 2003) and *Chlamydomonas reinhardtii* (Pérez-Pérez et al., 2017) and *Arabidopsis thaliana* (González et al., 2019) suggesting ALAD as an interaction partner of these plastidic reductants

The two paralogous *HEMB1* (AT1G69740) and *HEMB2* (AT1G44318) genes encode for the isoforms ALAD1 and ALAD2 in *A. thaliana*, respectively. ALAD1 is the dominant, possibly exclusive, variant in leaves as reported in Tang et al. (2012). Due to its essential importance, ALAD1 was used in our studies and for simplicity we refer to this isoform as ALAD throughout the manuscript. ALAD catalyzes the condensation of two molecules of 5-aminolevulinic acid (ALA) to porphobilinogen (PBG). ALA is formed in the rate-limiting step of TBS by glutamyl-tRNA reductase (GluTR) and glutamate 1-semialdehyde aminotransferase (GSAAT) (Tanaka and Tanaka, 2007). While the ALAD orthologues from human, animals, fungi, archaea and most bacteria recruit Zn^2+^-ions in their catalytic center (Jaffe, 2016), plant ALAD binds up to three Mg^2+^ cations, which are suggested to act (i) for catalysis in the active center, (ii) as an allosteric activator of the transition between different oligomeric ALAD forms and (iii) as an inhibitor during high Mg^2+^ concentration (Kervinen et al., 2000; Kokona et al., 2008; Jaffe, 2016). Interestingly, the studies of the posttranslational control of ALAD activity revealed a unique mechanism in TBS. Human and pea ALAD have been suggested to balance a regulatory equilibrium between a homohexameric, inactive form and a homooctameric, active form via the dissociation into dimeric intermediates. Thereby, ALAD interconverts between a “detached” and a “hugging” dimer (Kokona et al., 2008).

The plant ALAD activity depends on the presence of Mg^2+^ and differs pH-dependently with an enzymatic optimum in a slightly alkaline environment (Balange and Lambert, 1983; Kervinen et al., 2000). These two features considerably vary in chloroplasts during the diurnal plant growth. Moreover, ALAD activity is promoted by reducing agents, such as dithiothreitol (DTT) and the two isoforms TRX-f and TRX-m (Balange and Lambert, 1983). TRX-f turned out to be the more effective activator of ALAD than TRX-m (Balange and Lambert, 1983).

By a virus-induced gene silencing (VIGS) approach, the expression of the Arabidopsis *TRXm2, TRXm4* and *TRXm1* genes was simultaneously inactivated and the content of ALAD was decreased in the transgenic Arabidopsis lines compared to WT controls (Da et al., 2017). Similarly, the *ntrc* and *trxf1* seedlings contain less ALAD than wild type (WT), but not as much decreased as in TRX-m deficient plants (Wittmann et al., 2018). The lower content of ALAD correlates with its decreased *in vivo* activity in leaf extracts (Wittmann et al., 2018). As TRX also stimulates *in vitro* ALAD activity, it is intended to examine whether thiol-based redox regulation by TRX not only affects the stability of ALAD, but also its enzymatic activity. In continuation to our previous investigations on the redox control in TBS, we aimed to elucidate the possible thiol switches at the redox-sensitive Cys residues of *A. thaliana* ALAD and to identify the potential Cys residues for the thiol-based redox control. By using recombinant WT and Cys substitution mutants of ALAD their feasible redox switches were explored.

## Materials and methods

### Cloning, expression and purification of recombinant ALAD, TRX-f1 and TRX-m1

The full-length cDNA sequences of Arabidopsis the two ALAD (*HEMB1*; AT1G69740), TRX-f1 (AT3G02730) or TRX-m1 (AT1G03680) were cloned into the pET28a(+) expression vector (Novagen, Merck Millipore) without the coding sequences for their respective transit peptides. The lengths of the mature proteins were predicted by sequence analogy using the web tool ChloroP (Emanuelsson et al., 1999). The recombinant vectors were transformed into the *E. coli* Rosetta™ (DE3) strains (Novagen, Merck Millipore). The expression of *ALAD* in *E. coli* was induced by 1 mM isopropyl β-D-1-thiogalactopyranoside (IPTG) and continued under continuous shaking for 3-4 h at 37°C. The expression of TRX-f1 and TRX-m1 was induced by 0.4 mM IPTG and the cells were incubated for 3-4 h at 37°C. The N-terminal 6xHis-tagged fusion proteins were purified using Ni-NTA (nickel-nitrilotriacetic acid) agarose beads (Thermo Fisher Scientific), washed and released with imidazole containing buffer (50 mM Tris-HCl, pH 8, 300 mM NaCl, 10-250 mM imidazole). The buffer was finally exchanged to PBS (137 mM NaCl, 2.7 mM KCl, 10 mM Na_2_HPO_4_, 1.8 mM KH_2_PO_4_, pH 7.4) using Amicon^®^ Ultra-4 Centrifugal Filter Units (Merck-Millipore). Primers for the amplification of *A. thaliana* cDNA encoding for mature ALAD, TRX-f1 and TRX-m1 and for site directed mutagenesis PCR to replace each Cys of ALAD with Ser are found in **Supplementary Tables 1**, **2**.

### Site-directed mutagenesis PCR

To obtain the recombinant ALAD(Cys➔Ser) mutants, site directed mutagenesis PCR was performed with the pET28a(ALAD) vector as template. The protocol of Laible and Boonrod (2009) was followed to obtain the mutagenized ALAD sequences in the pET28a expression vector. The nucleotide exchanges in the *HEMB1* sequence were confirmed by sequencing.

### Gel-shift assays of AtALAD under reducing and oxidizing conditions

The concentration of the heterologously expressed proteins was measured using a BCA protein assay kit (Thermo Fisher Scientific) and confirmed by sodium dodecyl sulfate-polyacrylamide gel electrophoresis (SDS-PAGE) with a bovine serum albumin (BSA) gradient. For each reaction, recombinant ALAD was preincubated for 15 min at RT either with 10 mM DTT, 1 mM diamide or without redox agents (untreated). Then non-reducing Laemmli buffer (final concentration 50 mM Tris-HCl pH 6,8, 10% (v/v) glycerol, 2% (w/v) SDS, 0,02% (w/v) bromophenol blue) was added to the samples. The reduced/oxidized protein was separated by a 12% SDS-PAGE and subsequently blotted on nitrocellulose membranes. Finally, the membranes were probed with a 6×His-Tag specific antibody conjugated to HRP (Sigma-Aldrich), and the Clarity Western ECL Blotting Substrate (Bio-Rad) was used for protein detection with a CCD camera (INTAS Science Imaging Instruments).

For the reduction of intra- or intermolecular disulfide-bridges of ALAD by TRXs recombinant ALAD was incubated first for 30 min at 37°C with 1 mM diamide and subsequently dialyzed overnight at 4°C with a Slide-A-Lyzer™ MINI Dialysis Device (Thermo Fisher Scientific). The next day, the oxidized ALAD (1 µM) was incubated either with diamide (1 mM), DTT (0.1-10 mM) or 0.1 mM DTT together with recombinant TRX-f1/m1 (6.7 µM) for 30 min at 37°C under continuous shaking.

As control, ALAD was incubated with BSA (2 µM) in combination with DTT (0.1 mM). To exclude unspecific immune-reacting bands which could be generated by the purified recombinant TRX, the TRXs were also separately incubated with DTT (0.1 mM), but without ALAD. After TCA precipitation, the reduced Cys, which are accessible at the ALA surface, were blocked by incubation with 100 mM N-ethylmaleimide (NEM) for 1 h at room temperature (RT). Then the samples were diluted in non-reducing Laemmli buffer and separated by non-reducing 10% SDS-PAGE. The immune blotting is terminated with incubation of the membrane with a His-Tag specific antibody and the immune detection as described above.

### ALAD activity assay with recombinant protein and plant extracts

The ALAD activity assay was performed with total leaf extracts of the soluble fraction and recombinant proteins as previously described with small modifications (Wittmann et al., 2018).The preincubation recombinant ALAD under oxidizing (1 mM diamide) and reducing (10 mM DTT) conditions was performed in 50 µl volume for 15 min at 37°C under continuous shaking (800 rpm). The preincubated sample was transferred to 425 µl assay buffer and the reaction was started by addition of 25 µl ALA (100 mM). The reaction was stopped after 20 min incubation and continuous shaking at 37°C and the formation of PBG was photometrically quantified as described (Mauzerall and Granick, 1956). For the ALAD activity assay of plant extracts, leaf material was ground in liquid nitrogen, resuspended in extraction buffer (25 mM Tris-HCl, pH 8.2) and centrifuged. The supernatant was collected, the protein content quantified using the Pierce BCA Protein Assay Kit (Thermo Fisher Scientific) and used for the ALAD assay. The assay was performed by adding 1 Vol 2× reaction buffer (25 mM Tris-HCl pH 8.2, 10 mM ALA, 12 mM MgCl2, and the addition of 2 mM DTT for reducing conditions). Samples were incubated for 90 min at 37°C and constant shaking (600 rpm), before the reaction was stopped with 1 Vol 10% ice-cold TCA, 10 mM HgCl_2_, and porphobilinogen was quantified as described.

### Separation of native protein extracts by 2-D Blue native/SDS-PAGE

To analyze the *in vivo* ALAD complexes, a two dimensional Blue-Native/SDS polyacrylamide gel electrophoresis (2D-BN/SDS-PAGE) was performed. Leaf samples were homogenized and resuspended in PBS (137 mM NaCl, 2.7 mM KCl, 10 mM Na_2_HPO_4_, 1.8 mM KH_2_PO_4_, pH 7.4), the protein content was quantified by a BCA assay kit (Thermo Fisher) and the adjusted samples were subsequently mixed with 50BTH40G (50 mM Bis Tris/HCl pH 7.0, 40% (w/v) glycerol, 0.25 mg/ml protease inhibitor (Pefabloc, Merck Millipore) in a 1:1 ratio. The pretreatment of the samples with DTT (100 mM), MgCl_2_ (10 mM) or EDTA (10 mM) was performed at RT for 30 min. To solubilize the membrane proteins, 1% n-dodecyl-β-D-maltopyranoside (DDM) was added and the samples were incubated on ice for 5 min. After centrifugation, 0.1 vol of 10 × BN sample buffer [100 mM BisTris-HCl pH 7, 0.5 M 6-amino-caproic acid, 30% (v/v) glycerol), 0.05% (w/v) Serva Blue G)] was added to the supernatant. The samples were loaded onto a non-denaturing gradient gel (6 - 12.5% acrylamide) and the native complexes were separated in a first dimensional BN-PAGE according to Järvi et al. (2011).For the separation in the second dimension, the cutout gel slides were overlaid with 1 x SDS sample buffer (50 mM Tris-HCl pH 8, 100 mM DTT, 10% (v/v) glycerol, 2% (w/v) SDS, 0,02% (w/v) bromophenol blue) for 30 min. Then, the gel slides were placed horizontally on top of a 12% denaturing acrylamide gel and separated via SDS-PAGE. After immune-blotting to a nitrocellulose membrane, ALAD was immunologically detected by an ALAD-specific antibody (Wittmann et al., 2018).

### Structural prediction, bioinformatic work and statistical analysis

Protein structure prediction was performed via Alphafold2.3.2 (Jumper et al., 2021). Validation of the unstable regions of ALAD structure was conducted by using the predicted local distance difference test values (pLDDT, Guo et al., 2022). The further structure analysis was visualized by Chimerax (Pettersen et al., 2021).

In principle, always three independent biochemical repeats have been performed for each sample used for the presented experiments.

## Results

**Figure 1:**
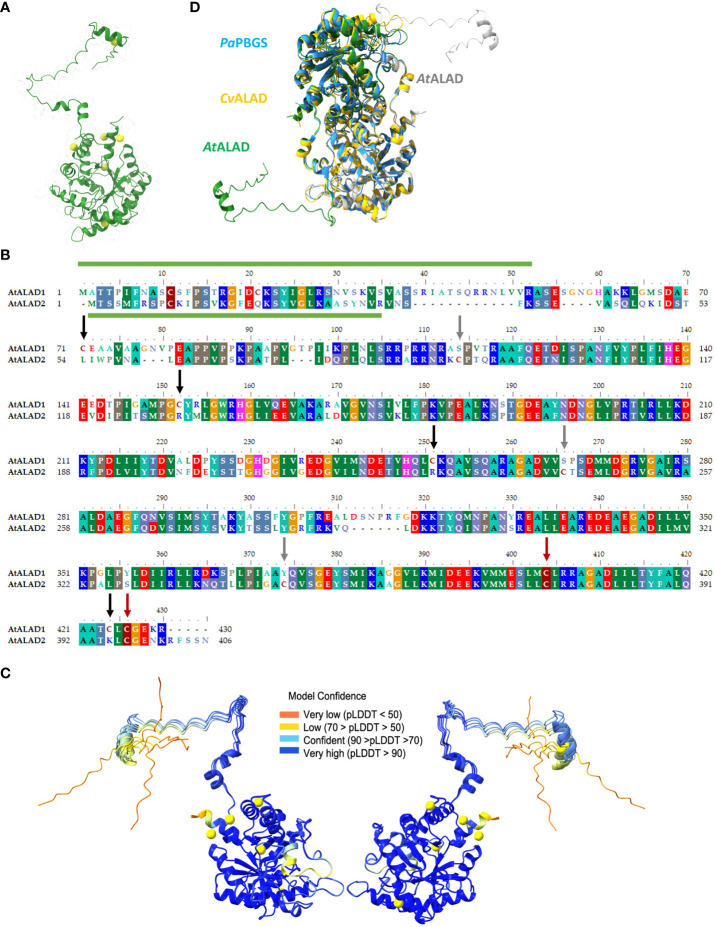
Structure and alignment of ALAD. **(A)** Structure prediction of mature ALAD1 (consisting of the sequence from Ala53 to Arg430) from Arabidopsis via AlphaFold (Jumper et al., 2021). The structure of the ALAD1 monomer was visualized via Chimerax (Pettersen et al., 2021) and all six cysteines (Cys) were highlighted in yellow. **(B)** Alignment the two ALAD isoforms of **(A)** thaliana encoded by HEMB1 (ALAD) and HEMB2 (ALAD2) performed with BioEdit (Hall, 1999). The conserved regions were underlaid with colors corresponding to the conserved amino acid residues. The appearance of conserved cysteines in the sequence was highlighted with burgundy, cysteines unique for ALAD with black and for ALAD2 with grey arrows. The predicted transit peptides include aa 1-52 for ALAD and aa 1-34 for ALAD2 (ChloroP) and are indicated with a green column above respectively under the sequence. **(C)** Representation of the probability of the modeled 3D structure of ALAD. The illustration shows the protein twice due to a horizontal 180° rotation. At the same time, this representation also reveals the areas of the ALAD structure whose spatial assignment is less predictable due to the greater dynamic flexibility. **(D)** Modelling of the *Arabidopsis thaliana* ALAD (AtALAD) dimer based on the data from the 3D structure of *Chlorobium vibrioforme* ALAD (CvALAD) in orange and *Pseudomonas aeruginosa* PBGS (PaPBGS) in blue. The upper AtALAD monomer is depicted in green and the other subunit in white.

Crystal structures of the Mg^2+^-dependent ALAD have been published from the gram-negative rod-shaped bacterium *Pseudomonas aeruginosa* (Frankenberg et al., 1999; Frere et al., 2002), and the photosynthetically active green sulfur bacterium *Chlorobium vibrioforme* (Coates et al., 2004; Coates et al., 2005). Both structures are strongly conserved. Based on these structures we modeled the *A. thaliana* ALAD (*At*ALAD) structure by means of AlphaFold [**Figure 1**, (Jumper et al., 2021)]. ALAD1 consists of 430 amino acid residues (including a 52 amino acid residue-long N-terminal, plastid transit peptide; from here on we call this isoform exclusively ALAD) and contains six Cys, whereby four of these are conserved in higher plants (Cys152, Cys251, Cys404, and Cys426, **Figure 1**). The monomeric structure of the mature ALAD comprises a flexible N-terminal arm and a globular peptide with a αβ-barrel domain, which include the active site between D220 and Y416 of AtALAD (predicted by Conserved Domain Database; Lu et al., 2020). It is worth noting that the variable N-terminal arm of the human was proposed to be decisive for the dynamic formation of the hugging and the detached dimer prior to the structural conversion into octameric and hexameric configuration (Breinig et al., 2003; Jaffe, 2016). The estimation of the probability of the modeled structure of ALAD is less predictable for some peptide domains due to their greater dynamic flexibility (**Figure 1**). This prediction-based analysis of the ALAD structure revealed that pLDDT values are low at the N-terminus, C-terminus and in the loop between Gly305 and Glu319, indicating a higher structural instability of these regions. These values also indicate the importance of the variable conformations in these regions for enzyme activity, oligomerisation and stability (Guo et al., 2022). The long mobile region of the N-terminus exhibits high flexibility and can swing around within a radius of about 40-60 Å, while the flexibility of the Gly305-Glu319 peptide region may be related to the entry and exit of Mg^2+^ ions and the ligands ALAD.

Based on the crystal structures of the two ALADs from *P. aeruginosa* and *C. vibrioforme*, we also modelled the structure of the dimeric ALAD from Arabidopsis. The three aligned structures showed a high degree of conformational similarity, with the exception of the N-terminal arm of the plant ALAD. Interestingly, the N-terminal arm of one ALAD molecule apparently interacts with the globule structure of the second monomer (**Figure 1**).

### TRX-f1 and TRX-m1 disassemble oligomeric structures of ALAD *in vitro*

**Figure 2:**
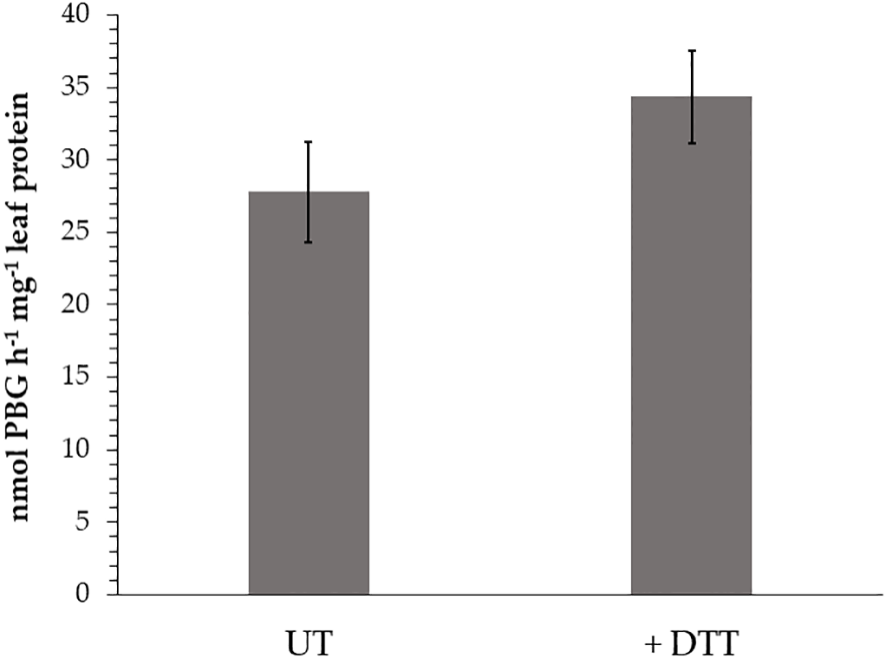
*In vitro* ALAD activity of wild-type (WT, Col-0) leaf extracts without (UT) or with (+ DTT) supplemented DTT (1 mM) to the assay buffer. The seedlings were grown for 3 weeks under short day conditions and 120 µmoles photon m^-2^ s^-1^ light intensity. Three biological replicates of each sample (SD) were used in the enzyme assays.

**Figure 3:**
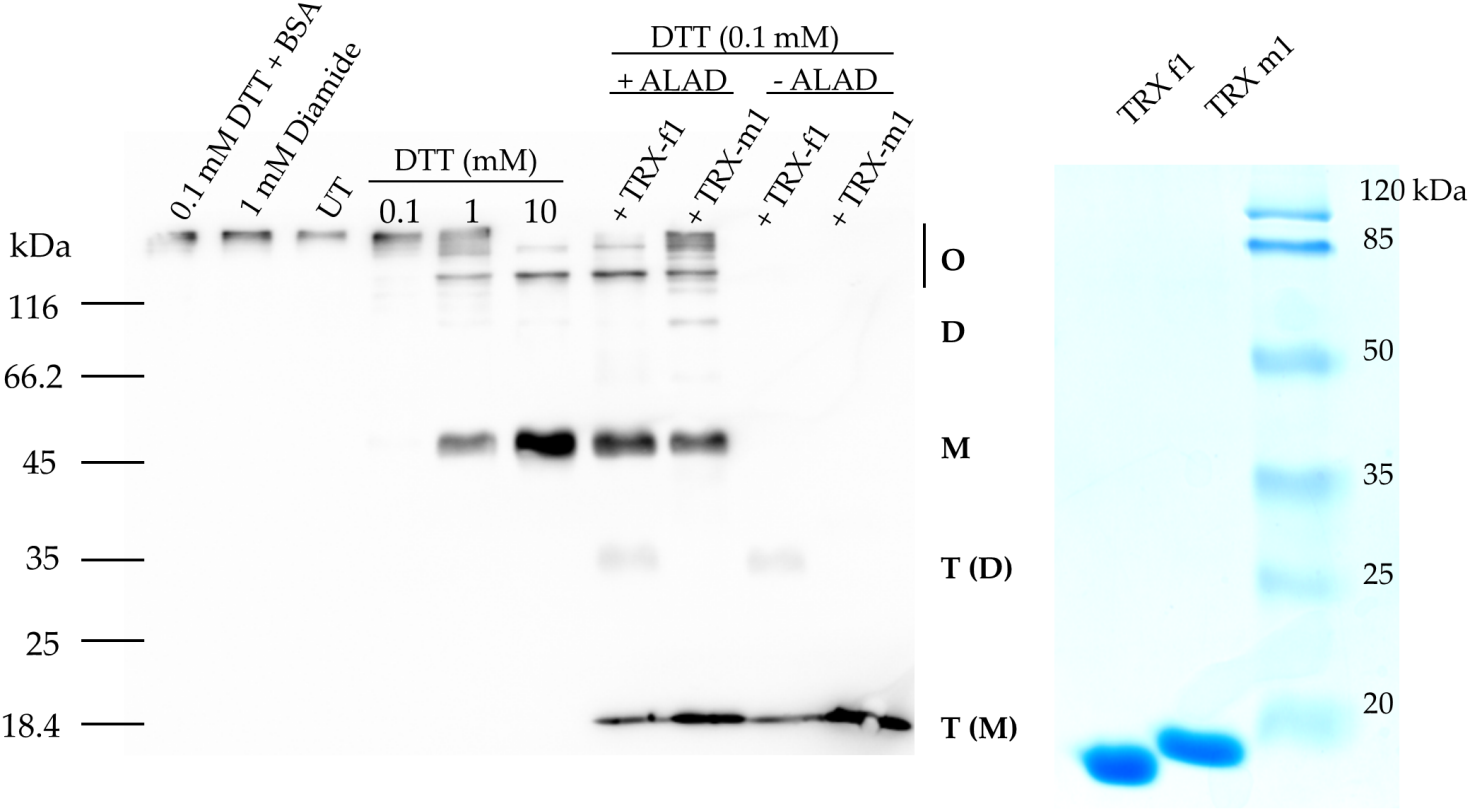
*In vitro* reduction of purified oxidized 6xHis-ALAD by means of increasing DTT con-centration and 6x-His-TRX-f1 and 6xHis TRX-m1. Oxidized ALAD (1 μM) was incubated for 30 min at room temperature with diamide, DTT, and the two TRX isoforms (6.7 μM) or BSA (2 μM). The free Cys residues of ALAD were blocked using NEM and the protein was separated in a non-reducing SDS-PAGE (10% acrylamide gel). Immune analysis revealed ALAD and TRXs using a His-tag specific antibody. Monomeric (M), dimeric (D) and oligomeric (O) forms of ALAD and TRX as monomer T(M) and dimer T(D) are displayed. UT, untreated purified ALAD sample.

First, the impact of additional supply of DTT to WT leaf extracts from three-week old Arabidopsis leaves for the ALAD activity was measured. ALAD activity could be stimulated by 20% with supplied DTT (**Figure 2**). This is consistent to in planta ALAD activities with and without DTT of leaf extracts from WT, *ntrc, trx f1*, and the *ntrc/trx f1* double mutant, which already reflect the redox dependency of ALAD stability and activity (Wittmann et al., 2018). Then, the *in vitro* effects of oxidizing and reducing agents on the degree of purified recombinant ALAD was initially confirmed. Partially purified ALAD (as shown in a representative experiment, see **Supplementary Figure 1**) was oxidized with 10 mM diazene dicarboxylic acid bis (N, N-dimethylamide) (diamide) to form disulfide bridges. Then, diamide was removed by dialysis. Upon addition of 0.1 mM DTT to the oxidized ALAD (1 µM), its initially formed oligomeric structure was not disassembled (**Figure 3**). ALAD was partially converted into the monomeric form upon higher concentrations of DTT. The higher the content of reductants, the more monomeric ALAD was detected (**Figure 3**). But even at the high concentration of 10 mM DTT, the high molecular weight ALAD oligomers were not entirely dismantled to monomers. In contrast, oxidizing conditions contributed to the assembly of ALAD dimers and high molecular weight oligomers. To investigate to which extent ALAD is reduced by TRX isoforms and how TRX affects the structure of ALAD, recombinant TRX-f1 and TRX-m1 isoforms (6,7 µM each) were supplied to ALAD together with 0.1 mM DTT. The reducing power of the two redox modifiers on ALAD was detectable, however, ALAD was not entirely converted to the monomeric form.

**Figure 4:**
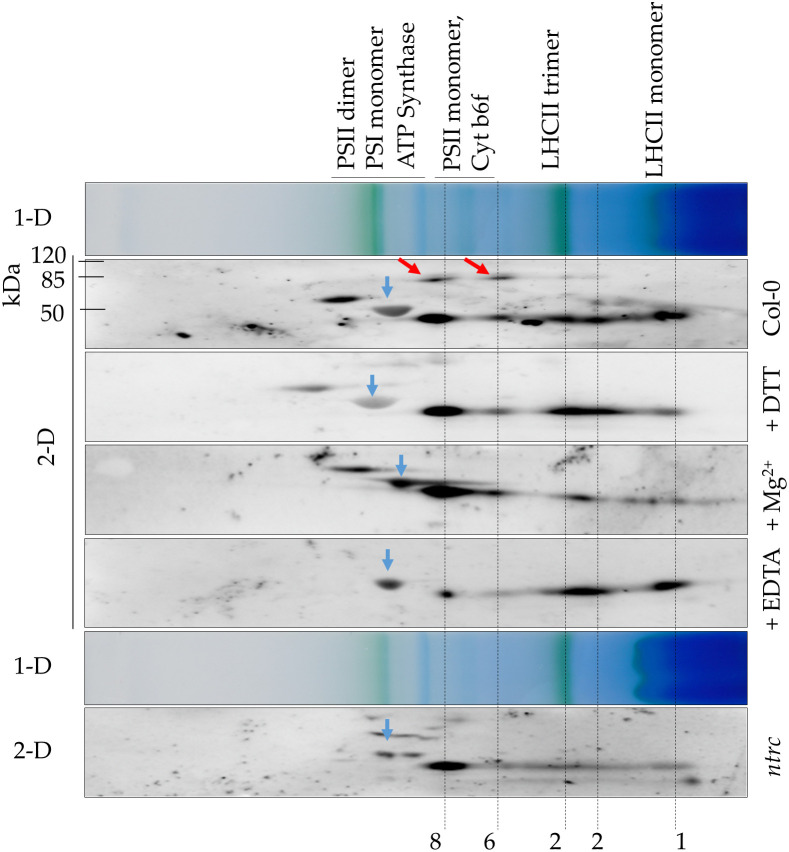
Predicted ALAD oligomerization of A. thaliana leaf extracts. Separation of protein extracts from WT (Col-0) and ntrc seedlings via 2D-BN/SDS-PAGE. Prior to electrophoretic separation, the native protein extracts were preincubated with 100 mM DTT (+DTT), 10 mM Mg2+ (+Mg2+) or 10 mM EDTA (+EDTA) or remained untreated (Col-0, ntrc). The extracts were solubilized with 1% DDM and separated on BN-PAGE. The proteins separated in the first dimension (1-D) were denatured by SDS and separated by SDS-PAGE (2-D). After the immune blot, ALAD was detected with the specific antibody. Red arrows: putative ALAD dimers, which were not denatured prior to separation in the denaturing SDS-PAGE. 1 monomer, 2 dimer, 6 hexamer, 8 octamer of ALAD. Blue arrows: large subunit of RuBisCO (~ 53 kDa). Photosynthetic protein complexes are indicated as size markers of the 1D BN gel (PSI 550 kDa, ATPase 300 kDa, LHC-Trimer 120 kDa); the molecular mass of standard proteins is indicated for the SDS gel. Three independent experiments have been performed for the samples and a representative data of one experiment are displayed.

While purified recombinant ALAD tends to assemble in oligomeric protein complexes, the *in planta* state of native ALAD in Arabidopsis leaf samples has not yet been determined. The oligomeric state of native ALAD was assessed by separation of soluble protein complexes of Arabidopsis total leaf extracts in a 2D-BN/SDS-PAGE. The soluble proteins of the leaf extracts were separated in a non-denaturing gradient BN-gel and subsequently in the second dimension with a SDS polyacrylamide gel. Immune analysis displayed ALAD with the specific antibody. **Figure 4** displays representative results from protein extracts of Arabidopsis WT and *ntrc* seedlings. According to the molecular size of abundant chloroplast-localized protein complexes, like the green-pigmented photosynthetic complexes of the thylakoid membranes, it is proposed that ALAD is separated in four differently sized states, which are assigned the monomer, dimer, and two oligomeric forms. Regarding the oligomeric structures of ALAD, we detected two additional immunoreactive high molecular weight spots in the WT in addition to the monomeric form. We hypothesize that these additional spots might be ALAD dimers (red arrows), indicating incomplete separation of ALAD subunits in SDS-PAGE. Comparing the 2D separation of ALAD with that of protein extracts supplemented with Mg^2+^, DTT or EDTA revealed that the ALAD high molecular weight oligomer migrates slightly slower than the PSII monomer and is more abundant upon addition of MgCl_2_ than in the *ntrc* extract or the EDTA-treated WT extract. This oligomer is not even dissolved upon addition of DTT suggesting that the treatment of WT extracts with reductants hardly change the ALAD multimeric structure under native conditions in plant extracts. EDTA pre-treatment removes Mg^2+^ ions in the ALAD structure resulting in the disassembly of the high molecular weight oligomer and the accumulation of more dimeric and monomeric ALAD conformations. Compared to the ALAD oligomerisation of the Col-0 control sample, the *ntrc* protein extract contains mainly the higher molecular weight oligomer, but to lesser extent the lower molecular weight oligomer and the dimeric variants.

In conclusion, Arabidopsis ALAD is detectable in different multimeric protein complexes. ALAD is assumed to form homomeric protein complexes of different sizes (**Figures 3**, **4**), similar to the homologous human, bacterial, and pea ALADs (Breinig et al., 2003; Kokona et al., 2008). The conformational changes were detected in non-denaturing gels and the quantitative differences of the oligomers were assessable in the second dimension of SDS PAGE. Moreover, the oligomerisation resembles the previous observations of Mg^2+^ and EDTA-dependent transitions between pea ALAD octamer and hexamer (Kokona et al., 2008; Jaffe and Lawrence, 2012). However, we cannot completely rule out the possibility that ALAD also interacts with other proteins in these higher molecular mass complexes.

### Cysteine-serine substitution mutants of recombinant ALAD

As previously shown, reductants such as TRX-f1 stimulate the catalytic activity of recombinant ALAD. It was proposed that one or more potential thiol-disulfide switches of ALAD modify its enzyme activity (Wittmann et al., 2018). The mature sequence of Arabidopsis ALAD contains six Cys residues (**Figure 1**). The two residues of AtALAD Cys251 and Cys404 are located in the active center of the enzyme (D220-Y416). Cys251 (CrCys204) and Cys404 (CrCys365) are also conserved in *Chlamydomonas reinhardtii* and AtCys404 (CvCys306) is also found in *Chlorobium vibrioforme*. The AtALAD2 sequence additionally contains three Cys residues at position 91, 243 and 345 (**Figure 1**). AtALAD2 shares only two Cys residues with AtALAD (Cys375 and Cys397 of ALAD2 are consistent to the homologous sites Cys404 and Cys426 of ALAD). ALAD likely represents the dominant isoform in Arabidopsis, as *HEMB2* expression is hardly detectable (Tang et al., 2012). Moreover, the *HEMB1* expression pattern characterizes ALAD as the plant-typical enzyme.

**Figure 5:**
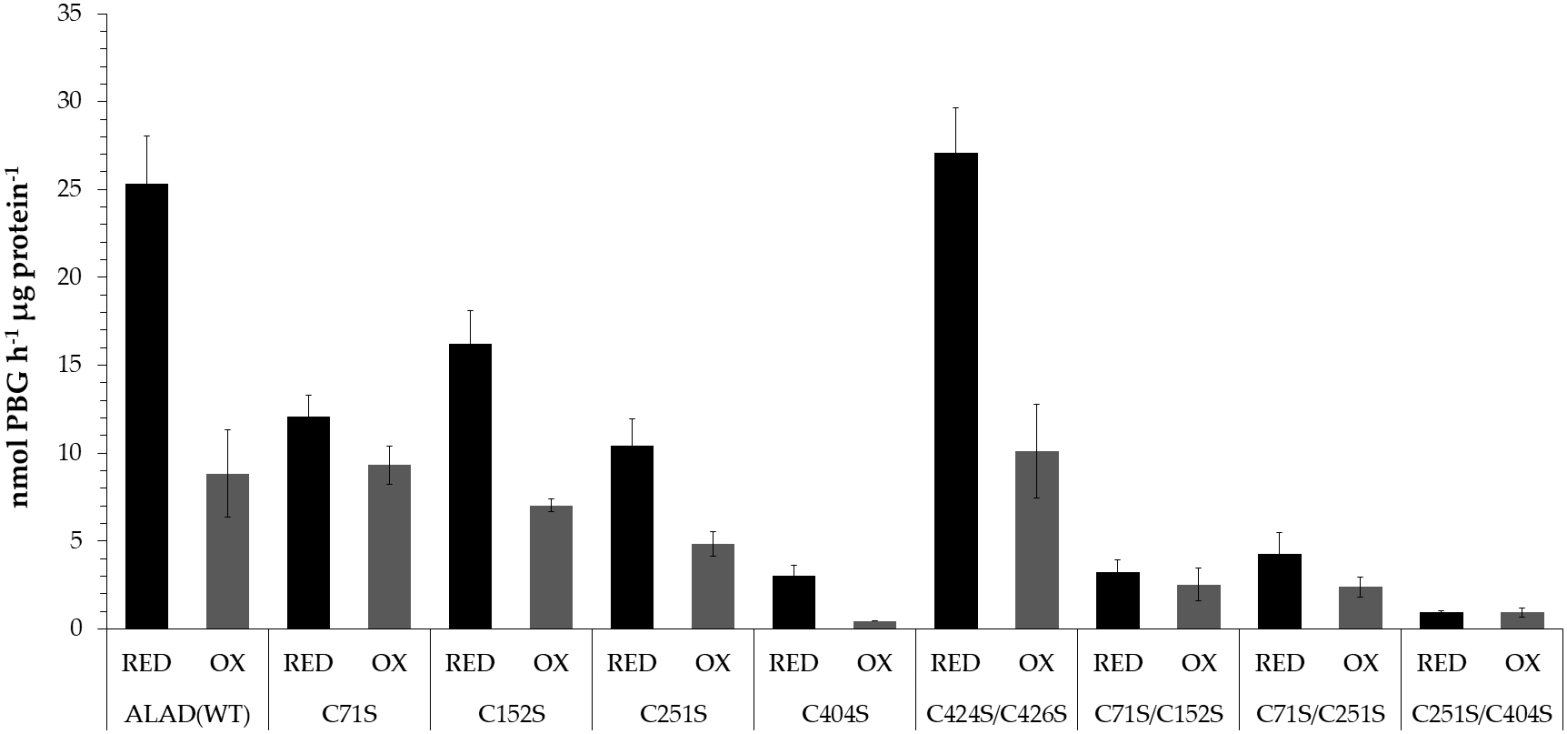
Activity of recombinant ALAD Cys➔Ser single and double substitution mutants after preincubation under oxidizing (1 mM diamide, OX) and reducing conditions (10 mM DTT, RED). The recombinant proteins were used from different purifications for the enzyme assays The preincubation took place for 30 min at 37°C. The PBG formed was detected photometrically using the Ehrlich reagent. The OD was measured at 555 nm and the PBG concentration was calculated using the molar extinction coefficient (Mauzerall and Granick, 1956). Results based on three replicates (SD).

As the mobility of maleimide labeled, recombinant ALAD in a non-reducing gel previously suggested, the Cys residues are responsive to oxidative disulfide bond formation (Wittmann et al., 2018) we intent to uncover these Cys residues, which contribute to the redox-dependent structural modifications of ALAD (Wittmann et al., 2018). It was expected that this attempt will disclose the redox-sensitive Cys residues of ALAD and succeed to assess the regulatory impact of thiol switches on oligomerisation, stability, and catalytic activity of ALAD. Six *ALAD* genes encoding the single Cys-Ser substitution mutants and four genes encoding double mutants were generated. The recombinant ALAD variants were produced in *E. coli* expression strains and subsequently purified and subjected to an enzyme assay after preincubation under oxidizing (1 mM diamide) and reducing conditions (10 mM DTT) (**Figure 5**).

Except the ALAD(C424S/C426S) double mutant, all Cys substitution mutants of ALA showed a lower enzyme activity than WT. The mutation at position 404 caused only less than 12% residual activity of the reduced variant indicating that C404 is positioned in the catalytic center of ALAD. The oxidized WT ALAD showed a drastically decreased enzyme activity (65% lower compared to the activity under reducing conditions). A weaker redox-sensitive change of catalytic activity was determined for the ALAD variants with a single substitution at the position Cys152 and Cys251. The oxidized form of these mutants displayed a 57% and 53% decreased activity, respectively, compared to the reduced activity indicating still a significant redox-dependent deactivation. Among the single substitution mutants only ALAD(C71S) showed a redox-insensitive enzyme activity. Among the ALAD double mutants, the C251S/C404S variant was almost entirely inactive, while ALAD(C424S/C426S) had a WT-like activity in the reduced and oxidized form (63% lower activity of the oxidized form relative to the reduced form). The two double mutants ALAD(C71S/C152S) and ALAD(C71S/C251S) possess also a rather redox-insensitive enzyme activity (**Figure 5**).

**Figure 6:**
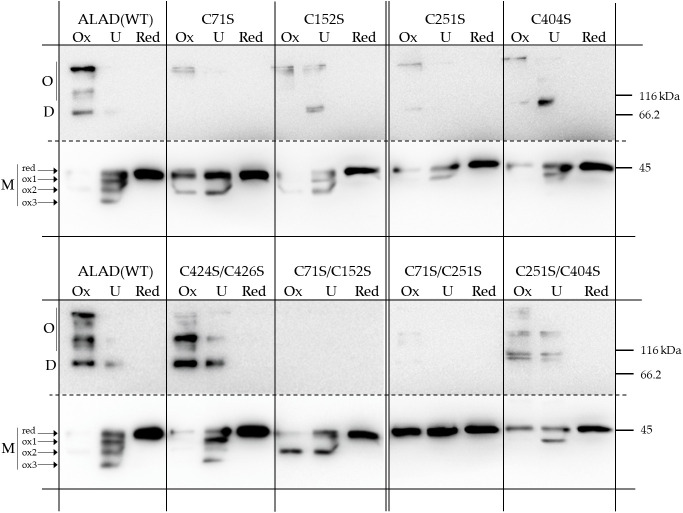
Redox-dependent oligomerisation of the purified 6xHis-ALAD with Cys➔Ser substitutions and the control ALAD(WT). The native proteins were preincubated either under oxidizing (OX, 1 mM diamide) or reducing conditions (RED, 10 mM DTT) for 15 min at RT before they were separated on a non-reducing SDS-PAGE (12% gel). UT = untreated. The immune-reacting bands were detected after immune blotting using a 6xHis-tag specific antibody. The upper part of the blotted membrane containing the multimeric forms (D, dimer; O oligomer) of ALAD is shown as a longer exposed image. The results based on three independent experiments (SD).

Subsequently, the recombinant ALAD substitution mutants were purified (as shown in a representative experiment, see **Supplementary Figure 1**) and analyzed for their ability to form intramolecular disulfide bonds and ALAD multimers. Preincubation with DTT leads to a sole protein band for recombinant WT and mutant ALAD variants in the non-reducing gels. Upon separation of the untreated ALAD samples, up to four monomeric redox states (red, ox1, ox2, ox3) were detectable for control ALAD (WT, **Figure 6**). Compared to the previous finding when only two oxidized forms were found (Wittmann et al., 2018), it is proposed that improved resolution in the SDS gel facilitated the specified protein separation. A similar WT-like protein pattern was observed for untreated ALAD(C424/C426) (as well as ALAD(C424S) and ALAD(C426S), not shown). Three redox forms (red, ox1, ox2) were detected for Arabidopsis ALAD(C152S), while only two monomeric redox states were displayed from ALAD(C71S) (red, ox2), ALAD(C251S) (red, ox1), ALAD(C71S/C152) (red, ox2), ALAD(C404S) (red, ox1) and ALAD(C251S/C404S) (red, ox1). Interestingly, ALAD(C71S/C251S) migrates only as a single reduced form (**Figure 6**).

Under oxidizing conditions, ALAD(WT) additionally shows formation of a dimer (approximately 90 kDa) and high molecular mass oligomers. The simultaneous mutation at positions Cys71 and Cys152 and Cys71 and Cys251 of ALAD prevented the formation of multimeric variants. With the exception of ALAD(C424S/C426S), all single ALAD mutants and the double mutant ALAD(C251S/C404S) show only sparse ALAD oligomerisation. Interestingly, the ALAD mutant with substituted Cys residues at position 71 and 251 showed the strongest restriction in the redox-dependent differentiation of the ALAD monomer. The mobility of the double mutant ALAD(C71S/C251S) under oxidizing and untreated condition is similar to that of the reduced monomer in the SDS gel, indicating a prevention of the formation of oxidized variants.

From these studies on redox-dependent oligomerisation, we deduce that the cysteine at position 71 is particularly important for the formation of the oxidized forms of the monomer of recombinant ALAD. We suggest that Cys71 localized in the flexible N-terminal arm of ALAD facilitates one or alternatively several disulfide bonds *in vitro*. We suggest that a swing of the N-terminal domain towards one of the Cys residues at the surface at the surface of the globular peptide enables a disulfide bonding (**Supplementary Figure 2**). In this figure, the possible thiol bonds of C71 with other cysteine side groups *in vitro* in monomeric ALAD are represented by simple linear linkage. Due to the presence of the rather mobile N-terminal loop region of more than 80 Å, Cys 71 has a more or less large possibility to swing to the thiol groups of the different Cysteines in the globular gestalt of ALAD. These potential intramolecular interactions do not necessarily occur in planta, as dimers could form rapidly *in vivo*, which would restrict the freedom of movement of the N-terminus according to the modeled structure of the AtALAD dimer (**Figure 1**). But, we suggest that the substitution of Cys71 compromises intramolecular disulfide bonding of the ALAD monomer.

In conclusion: Based on the biochemical data, the redox-dependent stability of ALAD di- and oligomerisation *in vitro* under oxidized conditions is strongly impaired in all single Cys-substitution mutants, in particular in the two double mutants ALAD(C71S/C152) and ALAD(C71S/C251), and to a lesser extent in ALAD(C251S/C404S). It is assumed that Cys71 has the strongest potential to form intramolecular disulfide bridges *in vitro*. If we look at the measurement of the ALAD enzyme activity, we see that a mutation of Cys404 drastically reduces the activity and that the ALAD(C71S/C152S) and ALAD(C71S/C251S) mutants have a rather redox-insensitive enzyme activity.

## Discussion

Plant TBS is light-dependently stimulated, while darkness suppresses the initial metabolic step, the synthesis of ALA, in response to the inactive light-operating POR (Richter et al., 2010). Thus, the metabolic flow between ALA synthesis and protochlorophyllide reduction is tightly regulated during day- and nighttime, but also balanced in response to varying environmental conditions (Brzezowski et al., 2015; Hou et al., 2019; Richter et al., 2019). An adjustment to the changing growth requirements is expected at almost every catalytic step, including ALAD, to obtain an instantaneous balance of proteostasis. This includes activity, stability, interaction with neighbor enzymes or oligomerisation and aggregation of the participating proteins. Redox-dependent control of ALAD activity and stability is the proposed mechanism at the step of PBG synthesis. Photosynthesis ensures an adequate environment of reducing conditions in the chloroplast, which may maintain ALAD active and stable. Light-induced transcriptional control of the *HEMB1* gene (Tang et al., 2012) is likely not involved in the rapid fine-tuning of ALAD activity.

It is interesting that native Arabidopsis extracts contain ALAD in protein complexes of different molecular sizes. AtALAD was found not only as a monomer and dimer, but also as at least two multimeric variants after separation in BN-polyacrylamide gels. Moreover, dimers were also disclosed in reducing and denatured SDS gels (**Figure 4**) and could be confirmed by modelling the ALAD structure based on the crystal structure of the two Mg-containing bacterial ALAD from *P. aeruginosa* and *C. vibrioforme* (**Figure 1**). In consistency, recombinant pea ALAD migrated also as two different multimers in non-denaturing gels. And it has been shown for human, bacterial, and pea ALAD that these homologous proteins are similar in their ability to form equilibrium between an octamer and a hexamer, with both oligomers formed via the formation of two distinct intermediate structures of dimers (Tang et al., 2006; Lawrence et al., 2009). As the recombinant AtALAD has high and a low molecular multimers (**Figures 3**-**4**, **6**) we hypothesize that these multimers may reflect the active and inactive ALAD variants, as previously reported (Jaffe, 2020). However, we failed to verify these ALAD multimers by other experimental approaches, such size exclusion chromatography by Sepharose S250 and Superose 6 Increase 10/300 GL columns or by sucrose density ultracentrifugation. Thus, it remains an intriguing question how the AtALAD multimer is assembled and whether there is a control between an active and an inactive (or less active) form of the oligomeric complexes.

Apart from the first indication of oligomeric ALAD structure, we intended to verify the impact of redox control on ALAD activity and oligomerisation and began to identify the Cys residues, which are assigned to be responsible for the redox-dependent modified enzyme activity and for the formation of the quaternary structure of ALAD. It is hypothesized that thiol switches at certain Cys residues play a crucial role for redox-dependent modulation of enzyme activity and stability. To predict a potential role of the different Cys residues in the intra- and intermolecular disulfide bonding of ALAD, the recombinant ALAD mutants with either one or two Cys ➔ Ser substitutions were analyzed.

The redox studies with recombinant ALAD substitution mutants substantiate that the *in vitro* oligomerisation of ALAD under oxidizing conditions depends on existing Cys residues at different positions in the ALAD structure. The extent of oligomerisation varied in the untreated and oxidized *in vitro* samples in comparison to the predominant portion of monomers under reducing conditions (**Figure 6**). In the SDS PAGE, the monomer of ALAD could be separated into differently migrating variants, which were distinguished between a reduced and three different oxidized forms (**Figure 6**). The distinctive protein mobility of three oxidized forms in the denaturing gel is proposed by modified configurations of ALAD likely due to the single and combined occurrence of disulfide bonds.

Considering the positions of Cys residues in the *A. thaliana* ALAD model (**Figure 1**) and their possibilities to form intramolecular disulfide bonds (**Supplementary Figure 2**), Cys71 in the flexible N-terminal arm of ALAD is the best candidate for the intramolecular and intermolecular thiol switches and shows the strongest potential to form intramolecular disulfide bridges *in vitro* (**Figure 6**). Based on the modified appearance of oxidized monomers of Cys-substituted mutants of ALAD, it can be proposed that Cys71 forms disulfide bonds with the two Cys residues at the positions 152 and 404. The assignment of the three differentially migrating ALAD bands under oxidizing conditions to either a fully oxidized ALAD variant or the mutant proteins containing one or two Cys substitution mutations is difficult and would not automatically correlate with the predicted possibility of a disulfide bond between two intramolecular Cys residues, as suggested by the structural model (**Supplementary Figure 2**).

However, since ALAD(C251S) and ALAD(C404) and the double mutant lack up to two of the oxidized ALAD bands in the SDS gel, we suggest that the remaining oxidized ALAD protein contains still a possible disulfide bond between Cys71 and Cys152 and migrates as the ox1 form. The ALAD ox2-variant could be linked to a disulfide-independent redox-modification of Cys251. Based on the structural model of ALAD (**Figure 1**) Cys251 can most likely not form an additional intramolecular disulfide bond with other Cys-thiol groups. Thus, Cys251 seems to be a potential site for another redox-dependent modification, such sulfenylation or an intermolecular disulfide bond. Redox-dependent proteomic studies revealed sulfenylated Arabidopsis ALAD (Akter et al., 2015). The ox3-variant could represent the combined disulfide bond formation between Cys71 and Cys152 or Cys404 and the redox modification on Cys251.

Incubation of ALAD under oxidizing conditions (CuCl_2_, diamide) or reducing conditions (DTT) leads to the modified enzyme activities (**Figure 3**; and Wittmann et al., 2018). The analysis of redox-dependent ALAD activity was advanced with the Cys-substitution mutants to correlate the finding on the redox-dependent oligomerisation of ALAD mutants with their redox-dependent catalytic activities. The oxidized forms of ALAD(WT) and C424S/C426S double mutant showed a 35% and 37% remaining activity of the reduced proteins, respectively. These two ALAD variants exhibited the strongest redox sensitivity of the enzyme activity. The activity assays exclude a putative role of the two 424/426 cysteines in redox-modulated activity. The ALAD(C424/C426) also showed a corresponding WT-like mobility in the non-reducing SDS-PAGE, excluding an impact on the oligomerisation of ALAD.

The loss of a thiol switch-dependent ALAD activity and modified structure become apparent for the mutant ALAD(C71S). This ALAD substitution mutant lost most of the redox sensitivity of ALAD activity, and their enzyme activity only slightly increased by 23% under reducing conditions. The loss of redox sensitivity can also be observed in the double mutants ALAD(C71S/C152S) and ALAD(C71S/C251S). However, the drastic reduction in enzyme activity of these two double mutants indicates the additional negative effects of each of the Cys substitutions on the overall activity of ALAD. The ALAD(C71S), ALAD(C152S) ALAD(C251S) show a 52%, 36% and 59% reduced activity, respectively, compared with ALAD(WT), while the double mutants exhibit a loss of 87% [ALAD(C71S/C152S)] and 83% [ALAD(C71S/C251S)] enzyme activity compared to ALAD(WT) (**Figure 5**). If the dimerization of ALAD requires an intermolecular disulfide bond between the Cys residues, then a thiol switch is preferentially proposed at position 404. In the model of the dimer structure both residues have a distance of 14,5 Å (**Supplementary Figure 3**). In the future, the question of how oxidizing conditions influence oligomerization, at least in *in vitro* conditions, and how oxidizing conditions lead to reduced activity should be resolved.

The plant ALAD sequences are characterized by the long N-terminal arm compared to the animal and bacterial homologs (**Figure 1**) and mostly possess an AtCys71 homolog indicating the significance of this N-terminal Cys residue, while pea or soybean ALAD and AtALAD2 miss this equivalent Cys residue. *A. thaliana* mature ALAD possesses six Cys residues, while only four Cys were found in pea ALAD. The homologous Cys residues at position 71 and 424 of AtALAD are missing in the pea ALAD. It will be instructive to examine assembly and stabilization of the pea and Arabidopsis ALAD homologs to unravel the control mechanism of oligomerization and the switch between inactive and active ALAD. In contrast to AtALAD, pea ALAD seems not to form intramolecular disulfide bonds between Cys residues of the globular protein (Kervinen et al., 2000). As no dimer formation of the pea ALAD(C326A) mutant (homologous to Cys426 in AtALAD) was observed it was predicted that this Cys residue of ALAD potentially is involved in intermolecular disulfide bonding, although ALAD(C326A) showed complete oligomerization and WT like enzyme activity (Kervinen et al., 2000). These observations resembled the unmodified catalytic activity and formation of multimers of the ALAD(C424S/C426S) variant in comparison to WT.

These current findings raise several issues, which need to be addressed in future studies. What could be the role of a redox-sensitive Cys71 for ALAD inactivation and oligomerization? It should be kept in mind that oligomerization of ALAD is verifiable in native gels under reducing and oxidizing conditions (**Figures 4**, **6**). It is evident that Mg^2+^ stabilizes the oligomers. The hindrance of oligomerization is only detectable when protein samples are subjected to denaturing gels. Thereby it became evident that ALAD(WT) requires reducing conditions to prevent oligomerization, while some ALAD Cys substitution mutants do not even assemble under oxidizing conditions.

Derived from our results, it is proposed that oxidizing conditions lead to intramolecular disulfide bonds formation in ALAD. In darkness, chloroplasts are characterized by oxidizing conditions and Mg^2+^ deficiency. These are preconditions for an adequate inactivation of ALAD. Given an oxidized environment in chloroplasts, inactive ALAD is achieved when assembly of ALAD monomers to octamers is attenuated. Then, AtALAD monomers could form an intramolecular disulfide bond and could assemble via dimers to the inactive hexamer. These predictions of a redox-dependent arrangement of ALAD subunits require further studies to verify the assembly and disassembly mechanism of AtALAD to build and stabilize the oligomeric ALAD structures. The structural arrangements between oligomers could be formed dependently or independently from redox-sensitive Cys residues, which are involved in intra- or intermolecular disulfide bridges.

In summary, the described Cys residues of ALAD seem to be not only relevant for the quaternary structure, but also for the modulation of the catalytic activity of ALAD during oxidized and reduced conditions. The presented data provide first indications for thiol-based redox regulation, which indicate a stimulatory effect on catalytic activity of ALAD under reducing conditions and the stabilization of oligomeric, inactive ALAD under oxidizing conditions. The formation and stability of the active ALAD octamer is supported by Mg^2+^. In future studies, the oligomerization and activity of Arabidopsis ALAD will be examined in planta by expression of the ALAD substitution mutants in the *hemb1* mutant background.

## Data availability statement

The original contributions presented in the study are included in the article/**Supplementary Material**. Further inquiries can be directed to the corresponding author.

## Author contributions

DW: Conceptualization, Investigation, Methodology, Writing – original draft. CW:. structual modelling, Writing -revised version, BG: Conceptualization, Funding acquisition, Supervision, Writing – original draft.
