# Supplementary material for: More indications for redox-sensitive cysteine residues of the Arabidopsis 5-aminolevulinate dehydratase
Source: Front Plant Sci. 2024 Jan 22;14:1294802. doi: 10.3389/fpls.2023.1294802 (PMC10839789; doi:10.3389/fpls.2023.1294802)
Supplement: Supplementary file 1 [file DataSheet_1.pdf]

Supplemental Table 1

Table 1. Primer for the amplification of A. thaliana cDNA encoding for mature ALAD, TRX-f1 and TRX-m1 for the ligation into the pet28a(+) expression vector by restriction digestion.

Description	Expression Vector	Sequence
ALAD fw (NheI)	pET28a(+)	GCTAGCGCTAGTGAATCCGGCAATGGA
ALAD rev (SacI)		GAGCTCCTACCGCTTCTCGCCGCACAA
TRXf1 fw (NdeI)	pET28a(+)	CATATGTGTAGCTTAGAAACCGTTAATG
TRXf1 rev (SacI)		GAGCTCTCATCCGGAAGCAGCAGACCTC
TRXm1 fw (NdeI)	pET28a(+)	CATATGCTATCTTCACTCTCGAAGAA
TRXm1 rev (HindIII)		AAGCTTTTACAAGAATTTGTTGATGC

Supplemental Table 2

Table 2. Primer for site directed mutagenesis PCR to replace each Cys of ALAD with Ser using pET28a(ALAD) as template.

ALAD(C71S)	fw	AGTGATGCTGAGTCTGAAGCTGCCGTT
	rev	AACGGCAGCTTCAGACTCAGCATCACT
ALAD(C152S)	fw	CTATGCCCGGTAGCTACAGACTTGG
	rev	CCAAGTCTGTAGCTACCGGGCATAG
ALAD(C251S)	fw	AGTACACCAGCTCAGCAAACAAGCTG
	rev	CAGCTTGTTTGCTGAGCTGGTGTACT
ALAD(C404S)	fw	AGTCACTGATGAGCTTACGCAGAGCT
	rev	AGCTCTGCGTAAGCTCATCAGTGACT
ALAD(C424S)	fw	CAAGCTGCTACTTCTTTGTGCGGCGAG
	rev	CTCGCCGCACAAAGAAGTAGCAGCTTG
ALAD(C426S)	fw	CTACTTGTTTGAGCGGCGAGAAGCG
	rev	CGCTTCTCGCCGCTCAAACAAGTAG
ALAD1(C424S/C426S)	fw	AGCTGCTACTTCTTTGAGCGGCGAGAAGC
	rev	GCTTCTCGCCGCTCAAAGAAGTAGCAGCT
