# Supplementary material for: More indications for redox-sensitive cysteine residues of the Arabidopsis 5-aminolevulinate dehydratase
Source: Front Plant Sci. 2024 Jan 22;14:1294802. doi: 10.3389/fpls.2023.1294802 (PMC10839789; doi:10.3389/fpls.2023.1294802)

Supplementary Materials

More indications for redox-sensitive cysteine residues of the Arabidopsis 5-aminolevulinate dehydratase

Daniel Wittmann and Bernhard Grimm

Supplementary Figure 1

Coumassie-staining of the purified variants of recombinant Arabidopsis 5-aminolevulinate dehydratase (ALAD) and recombinant thioredoxin f1 and m1 (TRX f1 and TRX m1). The images display a representative attempt to purify recombinant ALAD wild type (WT) and cysteine substitution mutants after expression in E. coli strains.

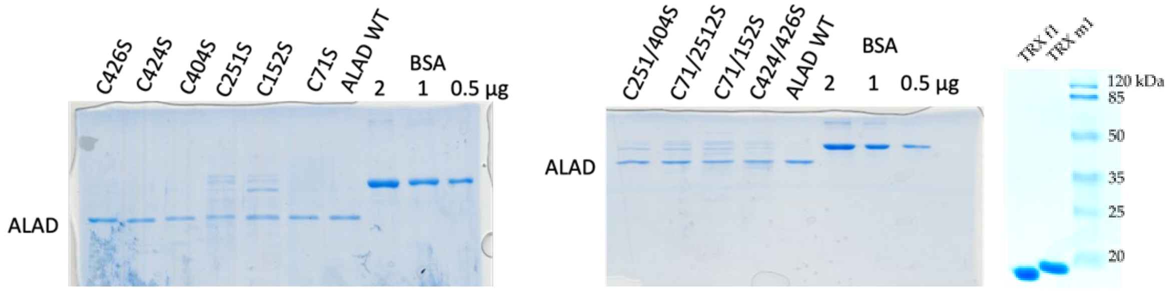

Supplementary Figure 2

In this figure, the possible thiol bonds of C71 with other cysteine side groups in vitro in monomeric ALAD are represented by simple dashes. Top left: Structure prediction of mature ALAD1 (consisting of the sequence from Ala53 to Arg430) from Arabidopsis via Alphafold (Jumper et al., 2021). The structure of the ALAD1 monomer was visualized via Chimerax (Petteresen et al., 2021) and all six cysteines (Cys) were highlighted in yellow. The other simplified representations refer to possible interactions of Cys71 with a thiol group of the other cysteines. These are purely speculative considerations of possible disulfide bonds. It can be seen which cysteines are more likely to undergo a thiol switch in vivo.

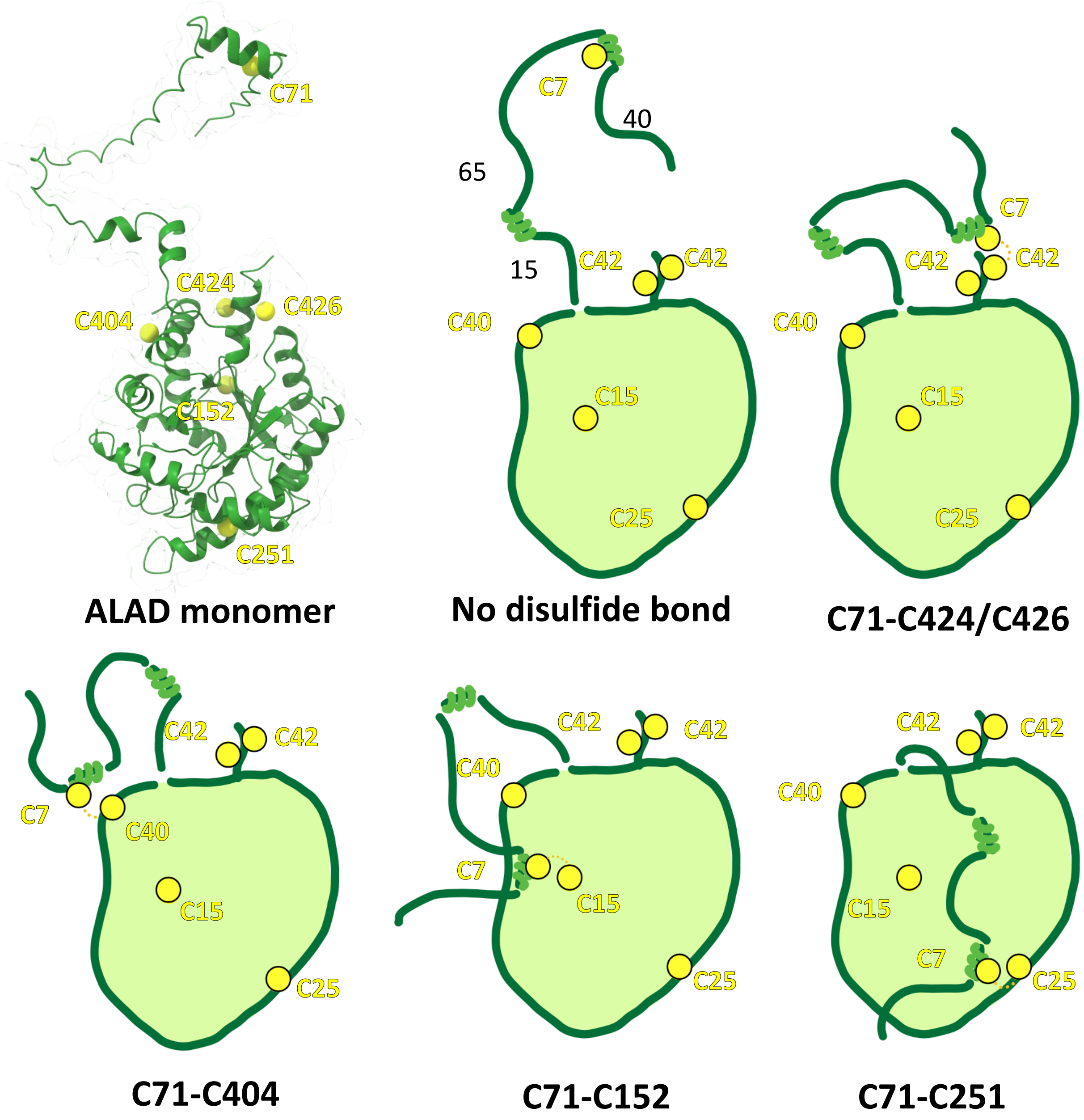

Supplementary Figure 3

Modeling of the dimeric Arabidopsis thaliana ALAD (AtALAD) structure obtained after structure prediction by AlphaFold. In the region of the opposite surfaces of both ALAD monomers, their two cysteines 404 have the highest potential probability of forming an intermolecular disulfide bond with their thiol side groups in the modeled dimer structure.

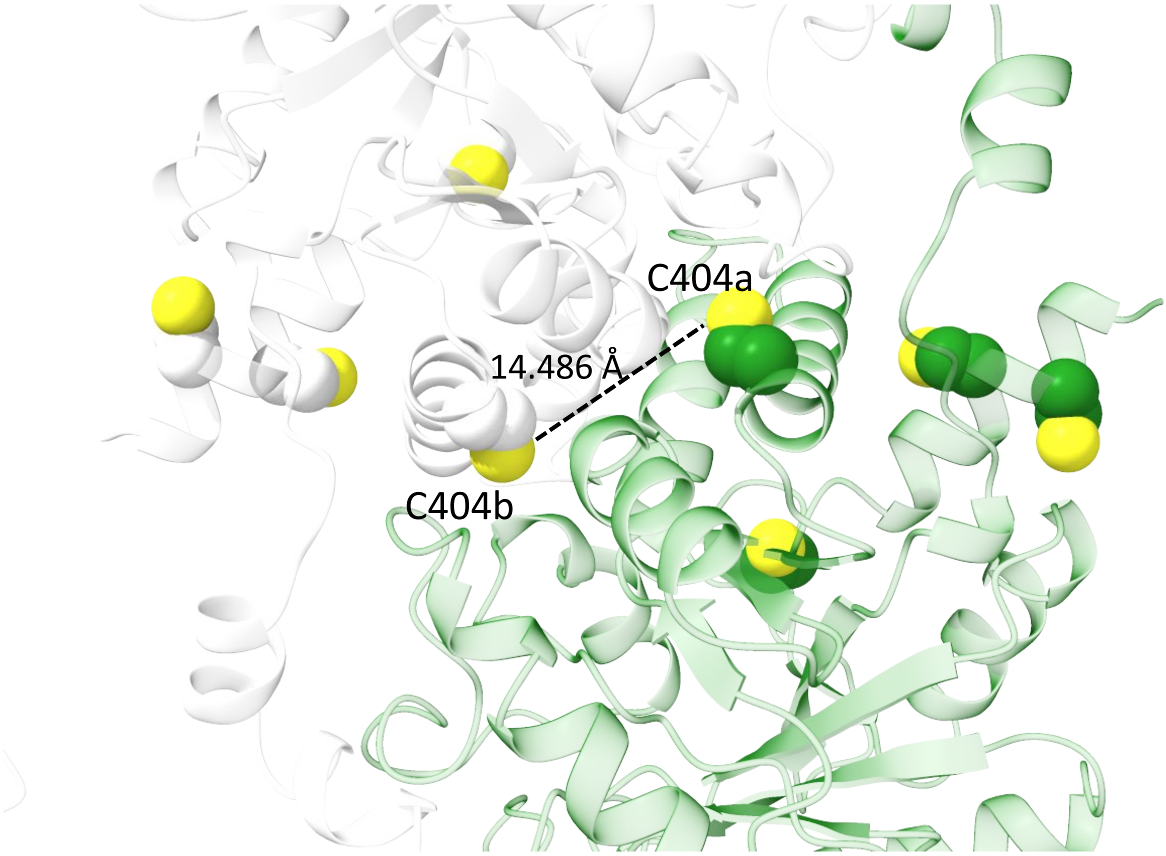

Supplement: Supplementary file 2 [file DataSheet_2.pdf]
